# Supplementary material for: Plasmodium falciparum subverts neutrophil function via host miR-451a loaded extracellular vesicles driving bacterial and fungal superinfection susceptibility
Source: PLoS Pathog. 2026 Sep 1;22(9):e1014533. doi: 10.1371/journal.ppat.1014533 (PMC13561428; doi:10.1371/journal.ppat.1014533)
Supplement: Supplementary file 1 [file ppat.1014533.s013.docx]

S1 Table. Number of differentially expressed genes in human neutrophils after different *in-vitro* stimulations

|  | **Up-regulated genes (%)** | **Down-regulated genes (%)** | **DE genes (%)** | **Unaffected genes** | **Expressed genes** |
| --- | --- | --- | --- | --- | --- |
| **EVs** | 3395 (25.1) | 3363 (24.8) | 6758 | 6757 | 13515 |
| **LPS** | 3832 (28.4) | 4016 (29.7) | 7848 | 5667 | 13515 |
| **LPSEV** | 3861 (28.6) | 3976 (29.4) | 7837 | 5678 | 13515 |
| **LPSEV vs Group lpsev** | 315 (2.3) | 196 (1.5) | 511 | 13004 | 13515 |
