# Supplementary material for: Plasmodium falciparum subverts neutrophil function via host miR-451a loaded extracellular vesicles driving bacterial and fungal superinfection susceptibility
Source: PLoS Pathog. 2026 Sep 1;22(9):e1014533. doi: 10.1371/journal.ppat.1014533 (PMC13561428; doi:10.1371/journal.ppat.1014533)
Supplement: Supplementary file 2 [file ppat.1014533.s014.docx]

S2 Table. Number of DEG in HL60miR451a after *in-vitro* stimulation with LPS at different time points

|  | **LPS treatment (min)** | **Up-regulated genes (%)** | **Down-regulated genes (%)** | **DE genes (%)** | **Unaffected genes** | **Expressed genes** |
| --- | --- | --- | --- | --- | --- | --- |
| **HL60miR451 vs HL60-GFP** | **0** | 3480 (24.4) | 3083 (21.6) | 6563 | 7713 | 14276 |
|  | **90** | 39 (0.3) | 105 (0.7) | 144 | 14132 | 14276 |
|  | **180** | 155 (1.8) | 261 (1.8) | 416 | 13860 | 14276 |
